# Applications of Acoustic Waves in Micro-Droplet Technologies

**DOI:** 10.3390/bios16090517

**Published:** 2026-09-13

**Authors:** Jianing Liu, Lijun Chen, Zheng Peng, Zachary B. Cluff, Pam Van Ry, Joseph Rich, Ruoyu Zhong

**Affiliations:** 1 School of Microelectronics, Shanghai University, Shanghai 200072, China; liujianing0307@shu.edu.cn (J.L.); chenlijun18@shu.edu.cn (L.C.); pengzheng@shu.edu.cn (Z.P.); 2 Department of Chemical and Biological Engineering, Brigham Young University, Provo, UT 84602, USA; cluffz01@student.byu.edu; 3 Department of Chemistry and Biochemistry, Brigham Young University, Provo, UT 84602, USA; pvanry@byu.edu; 4 Integrated Circuit Research Institute, Shanghai University, Shanghai 200072, China

**Keywords:** droplet microfluidics, acoustic waves, acoustofluidics, acoustic streaming, radiation force, droplet manipulation

## Abstract

Acoustofluidics has emerged as a powerful technique for the precise generation, sorting, manipulation, and stimulation of cell-laden droplets within emerging droplet microfluidic studies. Droplet acoustofluidics leverages various mechanisms, including acoustic radiation forces and acoustic streaming, to actively manipulate droplets non-invasively. The remarkable speed, precision, and biocompatibility of acoustic waves make it an ideal tool for droplet-based single-cell-level biomedical research. This review paper provides a comprehensive overview of recent advancements in droplet acoustofluidics technologies, highlighting their mechanisms, advantages, applications, and challenges. We have compiled the fundamental principles of acoustic waves and summarized major applications, including single-cell droplet generation, sorting, and active in-droplet manipulation. Despite the advances that current droplet acoustofluidics have achieved, we point out the challenges and give out our thoughts on future improvement directions. These insights will have important implications for advancing interdisciplinary research in cell mechanobiology, drug screening, cell sorting, and mechanophenotyping.

## 1. Introduction

Droplet microfluidics technology has revolutionized the field of microfluidic technology by enabling novel applications in single-cell-level biomedical engineering studies, with applications in cellular immunotherapy, cell interactions, drug screening, and single-cell dynamics [1]. Droplet microfluidic devices generate and manipulate sub-nanoliter-sized droplets [2,3,4], providing large-scale, individual units free from cross-contamination for single-cell isolation [5], cultivation [6], and analyses [5,7,8,9]. However, typical droplet microfluidics are limited by low cell encapsulation efficiency [10] and poor manipulation capability, which hinder their potential application in advanced biomedical research [1,10]. To overcome these constraints, a powerful, accurate, and contactless external force is required to actively manipulate the targeted droplets [2,5]. Several efforts (Table 1), such as electric [11,12,13], magnetic [14], optical, thermal [15], and acoustic forces [16,17,18,19], have been utilized to endow droplet microfluidics with unique features and capabilities [20,21]. With decades of development, various microfluidic manipulation techniques [22,23] have been developed and integrated with droplet microfluidics to emerge as a significant branch of micrometer-scale total analysis systems (microTAS). In 2017, Shang et al. [24] systemically reviewed the droplet microfluidics techniques, providing a general summary of droplet microfluidics procedures, related supporting technologies, and applications. In this review, we will focus on one branch, acoustic-powered droplet microfluidics, to give a comprehensive discussion of the unique advantages and challenges of acoustics in droplet microfluidics and microTAS.

Acoustic-powered droplet microfluidics, known as droplet acoustofluidics, is a major subcategory of droplet microTAS due to its unique advantages. Acoustofluidics (i.e., the fusion of acoustics and microfluidics) utilizes piezoelectric materials to generate acoustic forces, including acoustic radiation forces and acoustic streaming forces, via the mechanical propagation of acoustic waves. The most commonly used types of sound waves include surface acoustic wave (SAW) and bulk acoustic wave (BAW). SAW is precisely designed and can accurately control the applied acoustic force, while BAW has higher intensity and longer duration of action [25,26,27]. Due to their non-contact, precise, and biocompatible properties, acoustic waves can be applied for a variety of droplet-related applications, including droplet generation [28,29], sorting, manipulation, and sensing [30,31], which gives them great potential for advanced single-cell-level biomedical studies.

**Figure 1:**
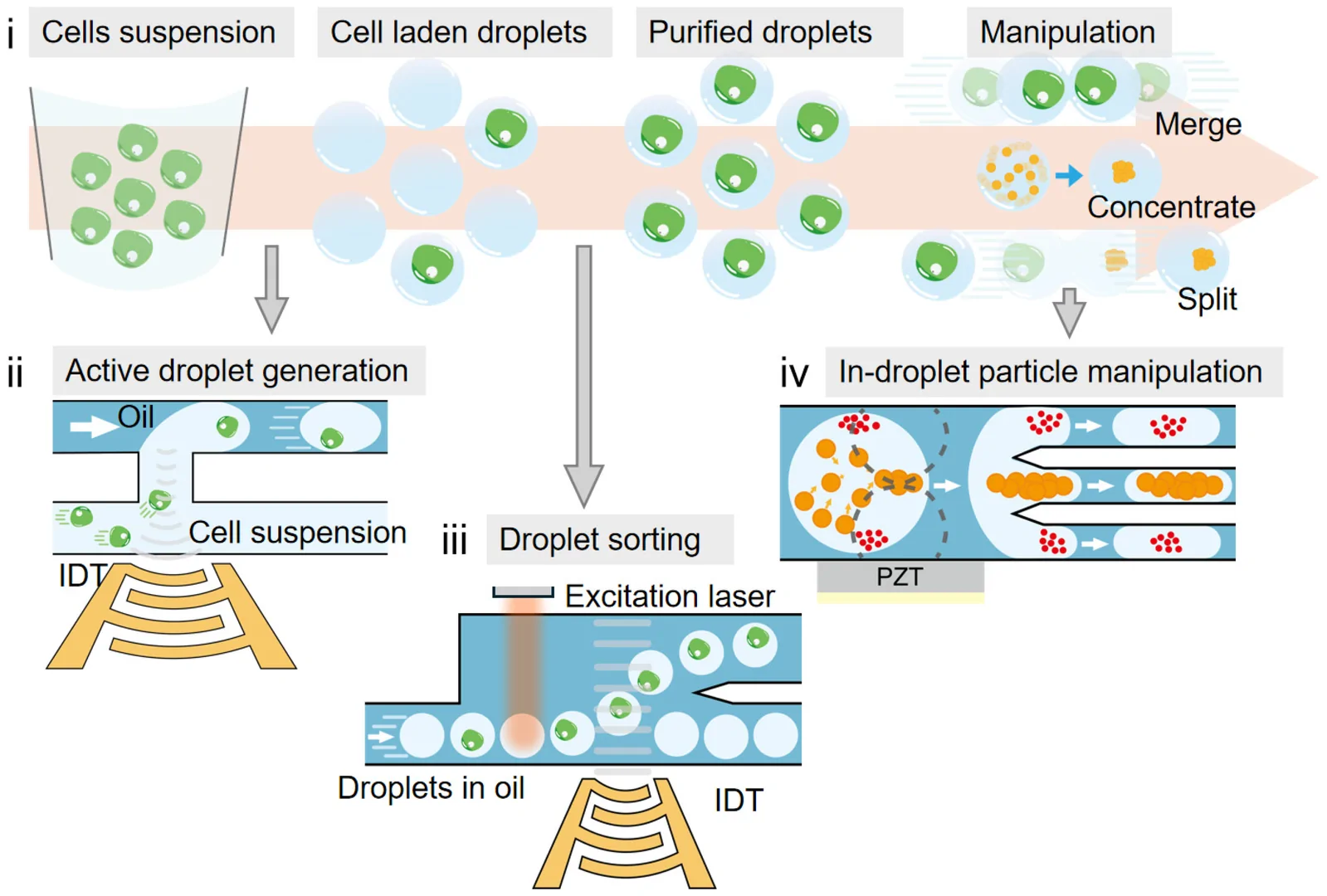
A diagram showing the process flow (**i**) and major applications (**ii**–**iv**) of droplet acoustofluidics: (**ii**) active droplet generation; (**iii**) droplet sorting; (**iv**) in-droplet particle manipulation.

Droplet acoustofluidics have been developed with two complementary aims: first, handling droplets that contain desired targets; second, manipulating targets within droplets. During the past few decades, acoustofluidics technology has played a significant role in all aspects of droplet microfluidics, spanning from droplet generation to detection (Figure 1). The developments and progress of droplet acoustofluidics technology will be discussed in detail in the following respects: active droplet generation, droplet sorting, and droplet manipulation.

**Table 1:** Comparison of various microfluidic methods [32].

	Acoustic Method	Electrical Method	Magnetic Method	Optical Method	Thermal Method
**Core driving force**	Acoustic radiation force, acoustic streaming	Continuous electrowetting	Magnetism	Optical gradient	Thermal dissipative forces, marangoni effect
**Signal activation**	No	No	Yes	No	No
**Biocompatibility**	High	Relatively low	Medium	Relatively low	Relatively low
**Flexibility**	High	High	Medium	High	Medium
**Typical** **operation**	Sorting; focusing; mixing; merge; arrangement	Sorting; driving; splitting; merge	Separation; combination; mixing	Trapping; transport; sorting	Driving; splitting; merge; trapping; temperature controlled reaction
**Primary** **limitations**	Complex equipment	Complex equipment; electrothermal damage	Magnetic bead carryover	Low throughput; expensive equipment	Thermal injury; bubble contamination

## 2. Principles of Droplet Acoustofluidics: From Waveforms to Driving Forces

### 2.1. Acoustic Wave Generation and Propagation: Surface vs. Bulk Waves

**Figure 2:**
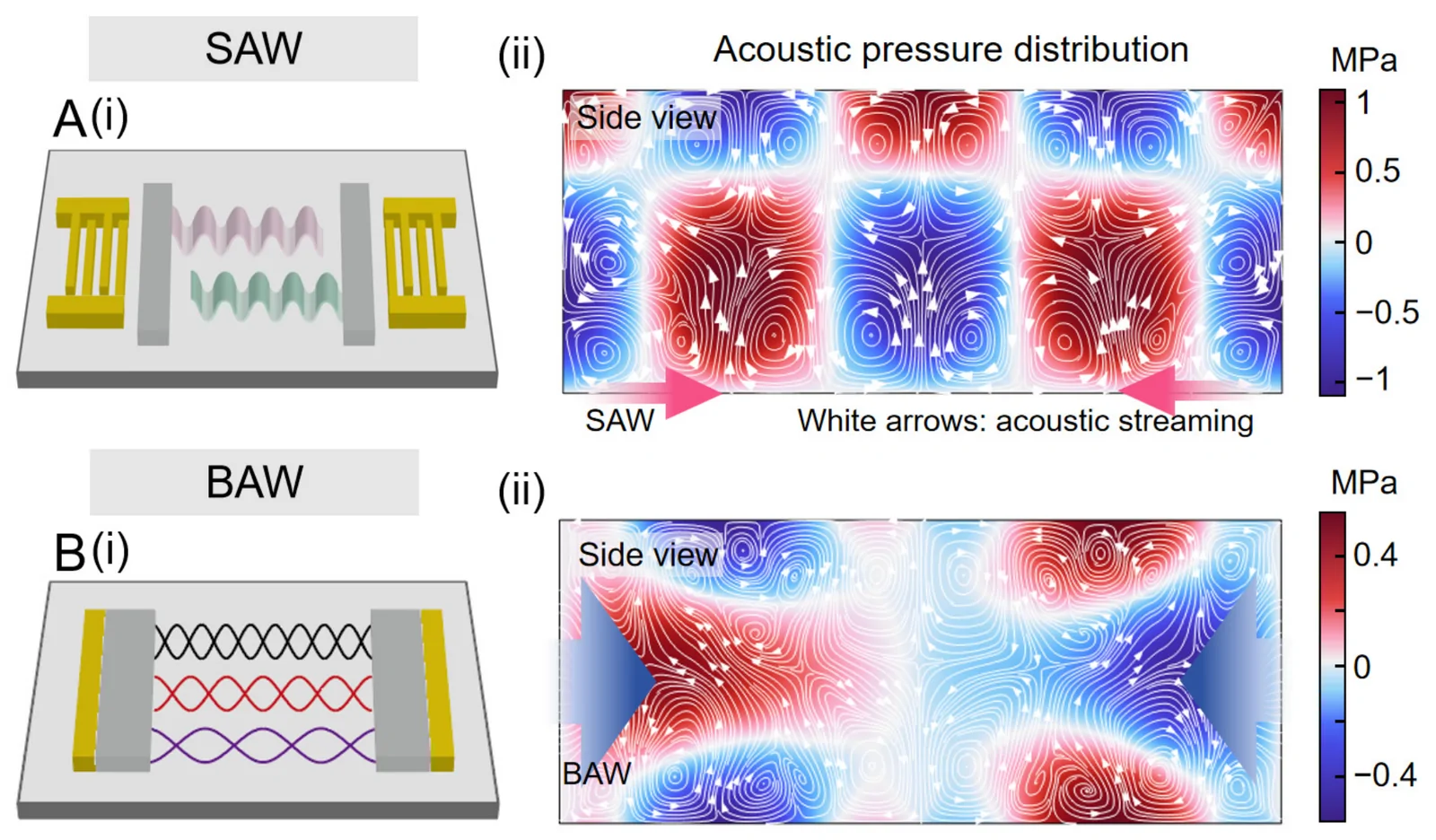
Basic working principles of the SAW/BAW-based chips. (**A**) Schematic of the SAW-based chip. (**i**) Typical structure and SAW waveforms [53]. (**ii**) Simulation results of the acoustic radiation force within the channel and acoustic streaming distribution; (**B**) schematic of the BAW-based chip. (**i**) Typical structure and BAW waveforms [53]. (**ii**) Acoustic radiation force and acoustic streaming distribution.

Based on the region where the acoustic wave energy is distributed and whether its propagation is affected by the boundaries of the medium, acoustic waves can be classified into surface acoustic waves (SAWs) or bulk acoustic waves (BAWs) (Figure 2) [33,34].

SAW is a wave in which energy is confined to the surface of a solid. Their frequencies are typically in the megahertz range, and their short wavelengths allow them to manipulate targets at the micrometer scale [35,36]. In 1965, R.M. White and F.W. Voltmer proposed that SAW arises from the interaction between a piezoelectric substrate and an interdigitated transducer (IDT) [37]. An IDT is a periodic array of metal electrodes fabricated on a piezoelectric substrate using photolithography (Figure 2A(i)). When the frequency of the alternating current matches the designed frequency of the IDT, it first induces periodic mechanical strain on the substrate surface via the inverse piezoelectric effect. The periodic structure then organizes discrete acoustic sources, causing the weak elastic waves excited by each finger to interfere along the propagation direction, ultimately forming a Rayleigh wave with concentrated energy and a stable waveform, named traveling surface acoustic wave (TSAW). Two traveling waves of the same frequency propagating in opposite directions form the standing surface acoustic wave (SSAW) (Figure 2A(ii)) [38].

SAW technology offers the advantages of highly focused acoustic fields and rapid response [39], but it also has some drawbacks. For example, the rapid energy decay makes it difficult to apply to large-volume samples. To address these issues, researchers have proposed several improvements to SAW-based chips. One direction is developing novel devices with coupled shear modes, leveraging the mutual coupling of multiple shear piezoelectric coefficients to serve high-frequency, wide-bandwidth SAW [40,41]. In addition, others focus on utilizing artificial acoustic microstructures to modulate the acoustic field, thereby improving uneven energy distributions [42,43,44]. However, some limitations remain, such as thermal effects that can cause damage to biological samples [45,46] and reliance on expensive micro- and nano-fabrication processes. Nevertheless, SAW-based chips have been applied to multiple fields including sorting, driving, and manipulation, proving their feasibility.

BAW is a mechanical wave that propagates within the bulk volume of an elastic medium, with frequencies reaching hundreds of megahertz (Figure 2B). Generating BAW requires exciting and utilizing the resonance phenomenon within a piezoelectric film. When an electrical signal is applied, the mechanical vibrations generated by the inverse piezoelectric effect propagate within the piezoelectric layer and reflect off its upper and lower boundaries (Figure 2B(i)). When the frequency of the applied electrical signal matches the natural mechanical resonance frequency of the piezoelectric transducer, the incident and reflected waves coherently interfere to form a strong standing wave. At this point, the vibrational energy is confined within the device, creating a stable and powerful BAW [47,48]. These waves reflect between the upper and lower walls of the microchannel, ultimately forming a stable, periodically distributed standing-wave field. Within this acoustic field, the point of lowest sound pressure is called a pressure node, while the point of highest sound pressure is called a pressure antinode [49].

Compared to SAW, BAW is better suited for high-frequency applications and requires simpler equipment; it also outperforms SAW in terms of temperature stability, insertion loss, and out-of-band attenuation. However, the uniform acoustic field distribution of BAW also means it lacks the ability to highly focus the acoustic waves (Figure 2B(ii)), resulting in some shortcomings in operational precision. To solve this issue, researchers tried different strategies to improve the accuracy of BAW, such as introducing microbubbles or localized resonant cavity structures to achieve localized acoustic field enhancement and precise control under low-frequency excitation [50,51], and utilizing piezoelectric phononic crystal plates that integrate transducers with artificial structures for trapping or suspending particles [52].

### 2.2. Acoustic Radiation Force

Acoustic radiation force is a steady-state force generated when acoustic waves transfer momentum to an object during propagation [54]. As early as 1902, L. Rayleigh [55] published a classic theory on acoustic resonance frequency. However, it was not until 1962 that L.P. Gorkov et al. [56] proposed an approximate expression more suitable for microscopic particles, which subsequently became the most commonly used theoretical tool in the field of acoustic manipulation. When acoustic waves encounter an object, they undergo complex interactions involving scattering, absorption, and re-radiation. During this process, the direction of motion in the surrounding medium differs when the acoustic wave compresses the object versus stretching it. These movements cannot cancel each other out within a single cycle, resulting in a phase difference between the sound pressure and the vibration velocity of the medium’s particles—this is the key mechanism behind the generation of acoustic radiation force [57].

The mechanism of acoustic radiation force varies significantly depending on the size of the object. When the object is much smaller than the wavelength, the acoustic field excites monopole and dipole scattering in the particle. The monopole arises from volume changes caused by the pressure, while the dipole stems from global oscillations driven by the pressure gradient. Together, these effects polarize the particle, which then couples with the background acoustic field, ultimately pushing the particle toward a stable region of the acoustic field [58]. It is worth noting that within this range, the magnitude of the acoustic radiation force is proportional to the cube of the particle diameter, meaning that even a slight increase in particle diameter leads to a dramatic increase in the force exerted on it [59,60]. However, when the particle size approaches or exceeds the acoustic wave wavelength, the effects of incidence and scattering must be taken into account. At this point, both the magnitude and direction of the acoustic radiation force oscillate with particle size, and the direction may even reverse [61,62,63].

### 2.3. Acoustic Streaming

Acoustic streaming refers to a stable macroscopic flow phenomenon driven by nonlinear effects resulting from energy attenuation when high-frequency acoustic waves propagate through a fluid [64,65,66]. Its theoretical foundation was established by Carl Eckart [67] as early as 1948. Its object of action is not objects within the fluid, but the fluid itself. Based on their mechanisms, acoustic streaming can be classified into two major categories: boundary-effect acoustic streaming and volume-effect acoustic streaming [68,69,70].

Volume-effect acoustic streaming is caused by volumetric attenuation as acoustic waves propagate within a fluid [67]. The flow structures of volume-effect acoustic streaming typically manifest as large-scale unidirectional or vortex flows and are commonly observed in high-intensity ultrasonic fields. When high-intensity acoustic waves propagate through a large-volume fluid, factors such as viscous dissipation and heat conduction within the fluid lead to a gradual loss of acoustic energy. This energy loss is converted into a steady-state volumetric force acting on the fluid volume elements [71,72]. Boundary-effect acoustic streaming, also known as the Rayleigh flow, is more common than the Eckart flow. Rayleigh flow typically occurs within the viscous boundary layer near solid walls [73]. When the acoustic wave acts on a solid boundary, due to the viscous adhesion between the fluid and the wall, the vibration velocity of the fluid approaches zero, while the velocity of fluid that is farther from the wall is above zero, creating a velocity gradient within the boundary layer. Under the influence of viscous dissipation, this velocity gradient is converted into a steady-state shear stress, which in turn drives the fluid to form a closed circulation structure [74].

## 3. Leading Platforms of Droplet Acoustofluidics Technologies: From Generation to Advanced Manipulation

### 3.1. Acoustic-Controlled Droplet Generation

#### 3.1.1. Physical Mechanisms and Theoretical Basis

Typical microfluidic droplet generation techniques primarily relied on passive methods, such as T-junctions, flow focusing, or co-flow structures to form droplets. Although these passive methods can produce highly monodisperse droplets, the droplet size and generation rate are predominantly governed by the channel geometry and the flow velocity. While adjusting the flow rate does offer some degree of post-fabrication tunability, it is inherently limited in its dynamic range and response time, and often couples the droplet size with the overall throughput. Consequently, achieving on-demand, real-time, and decoupled control over droplet dimensions remains unattainable with purely passive approaches. In addition, these methods are limited in handling atypical samples, such as high-viscose hydrogel or precisely encapsulating single cells [75,76,77]. To overcome these limitations, researchers explored active droplet generators. The key to the operation of active droplet generators lies in the formation of a continuous jet driven by pressure. They utilize external forces such as piezoelectric, acoustic, thermal bubble, or solenoid valves to apply periodic disturbances to the fluid, causing an imbalance in surface tension and forcing the jet to break periodically at fixed positions, thereby generating droplets of highly uniform size [78,79,80]. Active technologies can use external signals to control the droplet generation frequency and timing in real time, enabling true on-demand droplet generation. This makes them particularly suitable for applications such as inkjet printing, high-throughput digital microfluidic analysis, drug microsphere preparation, and advanced manufacturing [81,82].

An acoustic droplet generator is a device that employs precise physical forces exerted by high-frequency acoustic waves to realize non-contact liquid manipulation and on-demand droplet generation [83]. The entire process is dominated by two fundamental acoustic physical effects: acoustic radiation force and acoustic streaming [84]. Of these two core effects, the acoustic radiation force is the primary driving force [85]. During droplet generation, either by using IDT to excite SAW or by positioning a BAW transducer at a specific location, the acoustic radiation force can be precisely focused onto the liquid jet at the outlet [83,86]. By focusing an alternating perturbation on the liquid column, the column undergoes Rayleigh–Pratt instability, resulting in the formation of a separate droplet at the neck [87].

According to the theory of linear stability of cylindrical jets, the quantitative evolution of this instability process is strictly governed by the dispersion relation [88]. For viscous liquids actually involved in breakage, let the time-dependent perturbation be denoted as $e^{st}$ (where s is a complex growth rate, and its real part represents the perturbation growth rate), the axial wavenumber of the perturbation be k (which originates from the spatially periodic sinusoidal perturbation imposed on the axial interface of the liquid column), and the radius of the liquid column be a. Then, the relationship between the perturbation growth rate and the physical property parameters is given by

$${\left(s+2\nu k^{2}\right)}^{2}=\frac{\sigma k}{\rho a^{2}}\cdot \frac{I_{1}(\mathrm{ka})}{I_{0}(\mathrm{ka})}\cdot (1-k^{2}a^{2})+4\nu^{2}k^{4}.$$

Here, σ is the surface tension coefficient, ρ is the density, ν = η/ρ is the kinematic viscosity, and $I_{0}$ and $I_{1}$ are the first-kind modified Bessel functions, which reflect the radial decay distribution of the disturbance. This equation mathematically defines the critical criterion for instability: only for long-wavelength perturbations satisfying ka<1 (equivalent to a perturbation wavelength λ>2πa) is the first term on the right-hand side of the equation positive, resulting in s having a positive real part and providing the physical prerequisite for the liquid column to grow exponentially and rupture; conversely, if ka>1, all perturbations decay exponentially, and the liquid column remains stable.

The acoustic radiation force actively and selectively excites the fastest-growing mode that satisfies ka<1 (corresponding to ka≈0.697 in classical Rayleigh theory) by modulating the energy and frequency of the input perturbation wave. The value of s in the dispersion relation directly determines the time required for the neck radius to contract until fracture occurs. Therefore, by precisely adjusting the perturbation frequency and amplitude through acoustic parameters, one essentially controls the initial boundary conditions and the effective wave number k in this dispersion equation, thereby achieving high-precision digital programming of the fracture timing and droplet volume [89]. Compared to the acoustic radiation force, the acoustic streaming effect primarily serves a supplementary and enhancing role. Within the channel, the acoustic streaming effect can effectively accelerate the mixing of substances in the liquid, ensuring the uniformity of the reaction process. At the moment when a droplet is about to split from the liquid column, the shear force generated by the acoustic streaming also ensures that the droplet detaches from the liquid column quickly and cleanly, significantly improving the success rate of droplet generation. Furthermore, in certain specific designs, the shear force generated by the acoustic streaming itself can serve as the primary driving force for droplet separation [84].

#### 3.1.2. Technical Evolution and Performance Metrics

**Figure 3:**
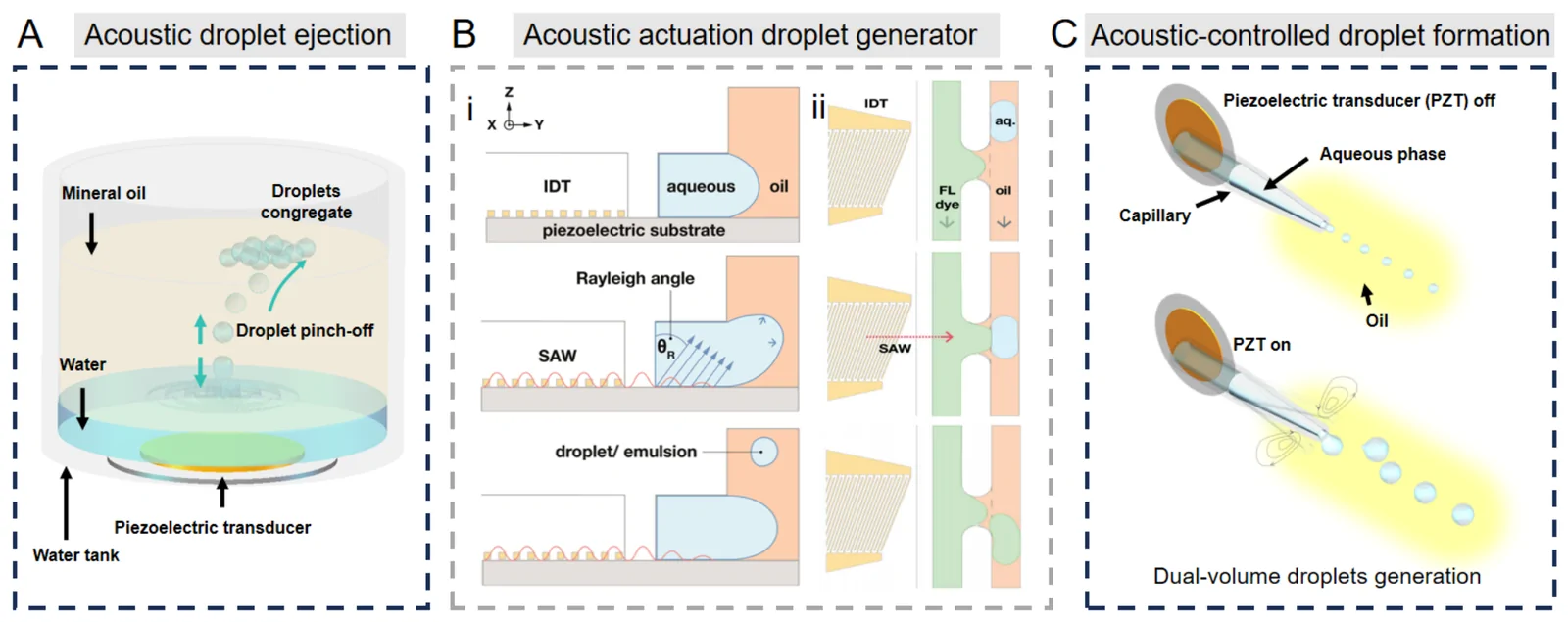
A diagram showing the principle and categories of acoustic droplet generation methods. (**A**) Acoustic droplet ejection, the core process of droplet generation [86]; (**B**) acoustic actuation droplet generator, droplet generation based on TSAW (**i**) and droplet generation combining TSAW and fluorescence technology (**ii**) [29]; (**C**) acoustic-controlled droplet formation, ultrasound-driven tip capillary droplet generator [98].

Based on these physical mechanisms, researchers demonstrated the feasibility of using SAW to generate single droplets on demand (Table 2). In 2013, D. J. Collins et al. [90] advanced droplet generation to the picoliter scale and were able to pre-concentrate and encapsulate particles or cells during the generation process, marking the initial emergence of acoustic droplet generation. Subsequently, L. Schmid et al. [91,92] further integrated SAW into traditional passive microfluidic architectures and successively applied it to two types of droplet generators—flow focusing and T-junction devices—enabling real-time electronic control of droplet size. This work remarks that droplet volume can be precisely controlled even when flow velocity and channel geometry remain unchanged. Key performance metrics of acoustic droplet generation have also seen significant improvements over time. Regarding lower volume limit control, in 2016, J. C. Brenker et al. [93] utilized the pressure source generated by high-frequency SAW to reduce droplet sizes to the femtoliter scale, and systematically elucidated the relationship between different acoustic drive parameters and the corresponding droplet-generation mechanisms. As for the driving principle, the introduction of focused surface acoustic waves (FSAWs) also brings a revolution. According to a 2019 study by S. Jin et al. [94], droplet detachment no longer relies primarily on fluid shear forces but instead directly depends on the strength of the focused acoustic forces. Subsequently, BAW technology was also brought into the field. In 2023, Y. Zhou et al. [86] utilized the acoustic forces excited by terahertz BAW at the oil–water interface to sever the liquid column, enabling droplet size control spanning four orders of magnitude even without the involvement of nozzles or microchannels (Figure 3A).

The practical demands of biomedical applications have, in turn, accelerated advancements in acoustic droplet generation technology. For instance, in single-cell encapsulation, traditional passive methods are governed by a Poisson distribution, which usually generates a high proportion of empty or multi-cell droplets, hindering the occupancy of desired single-cell droplets. In 2020, K. Mutafopulos et al. [29] utilized TSAW to achieve fluorescence-activated on-demand generation (Figure 3B(i,ii)). Under acoustic control, single-cell encapsulation has become a primary outcome. Significant progress has also been made in SAW-assisted schemes for handling high-viscosity dispersed-phase fluids. In 2022, S. Zhao et al. [95] provided a systematic description of the generation mechanism of high-viscosity microdroplets through precise acoustic control and achieved effective intervention. At the same time, they extended the application of acoustic methods to the generation and arrangement of liquid metal microdroplets [96]. In previous studies, high-surface-tension fluids were difficult to disperse uniformly and did not readily form ordered arrays; the incorporation of acoustic methods has addressed these issues to some extent. Simplification and innovation in device structures constitute another avenue of exploration for acoustic droplet generation technology. In 2021, Z. He et al. [97] proposed a vibrating sharp-tipped capillary scheme that maintains efficient droplet production solely through acoustic streaming, completely eliminating the need for an external pressure source, while allowing real-time adjustment of droplet size over a wide range. In 2026, Q. Zhang et al. [98] further incorporated programmable design, enabling the on-demand generation of sequences of droplets with varying volumes, thereby accelerating the practical implementation of portable, low-cost droplet generation systems (Figure 3C).

**Table 2:** Generational chart of droplet generation controlled by acoustofluidics.

Author	Year	Main Content	
D. J. Collins, T. Alan, K. Helmerson	2013	Traditional SAW devices operating within the MHz frequency range (48.4 MHz) enable on-demand, programmable generation of pL-scale droplets. Enables pre-concentration and encapsulation of particles or cells during the droplet formation process.	[90]
L. Schmid, T. Franke	2013	SAW is integrated into the flow-focusing droplet generator. The operating frequency is raised to 161 MHz and the power is limited to 200–800 mW; the droplet generation rate is increased. Enables real-time and dynamic control of droplet size.	[91]
L. Schmid, T. Franke	2014	SAW is integrated into the T-junction droplet generator, operating at a power of only 200 mW. Achieves rapid and precise electronic control of droplet size. Viscosity and surface tension do not affect droplet generation.	[92]
J. C. Brenker, D. J. Collins	2016	SAW is integrated into the droplet generator with a quasi-3D nozzle structure. The droplet generation size is elevated to 200 fL. The relationship between different generation mechanisms and droplet sizes is systematically revealed.	[93]
S. Jin, X. Wei, Z. Liu	2019	A novel MHz-range droplet generation method based on FSAW. Eliminates the reliance on fluid shear force. Acoustic energy acts as the sole driving force for μm-sized droplet detachment.	[94]
K. Mutafopulos, P. J. Lu	2020	Realization of MHz-level fluorescence-activated on-demand droplet generation based on TSAW. Equipped with a fluorescence module for selective control. Breaks the Poisson distribution limitation. Enables single-cell encapsulation as the dominant outcome.	[29]
Z. He, J. Wang, B. J. Fike	2021	First induced acoustic streaming to vibrate the sharp-tip capillary for droplet generation. Very low complexity is realized, operation at ~97 kHz is adopted, power consumption below 60 mW is maintained, and no external pressure source is required. Real-time modulation of droplet size over an ultra-wide range from 163 fL to 151 nL.	[97]
S. Zhao, Z. Liu, J. Wang	2022	Achieves high-viscosity microdroplet generation by MHz-level SAW. Overcomes the bottlenecks of unstable interface breakup. Great size controllability.	[95]
Y. Zhou, M. He, H. Zhang	2023	A nozzle-free droplet generation method based on GHz BAW. Droplets are generated at the oil–water interface merely by acoustic energy. Regulation of droplet size spanning from 2 μm to 1800 μm.	[86]
S. Zhao, Z. Liu, N. Zheng	2025	SAW is applied to the generation and spatial modulation of liquid metal microdroplets. Solves the challenge of achieving uniform dispersion. Ordered array formation for fluids with high interfacial tension.	[96]
Q. Zhang, L. Ran, G. Li	2026	Ultrasound-driven sharp-tip capillary droplet generation. Programmable on-demand generation of multi-volume droplet sequences over a range of 6 pL to 29 nL.	[98]

Compared with other active droplet generators, the core advantages of acoustic droplet generators lie in their high biocompatibility and versatility. Unlike electrically driven systems, they do not rely on specific fluid properties but only require a difference in acoustic impedance. They can operate with aqueous phases, oil phases, and even complex biological fluids, greatly expanding their range of applications [25]. Compared to magnetic drive systems, they do not require the integration of magnetic components, thereby preserving the purity and integrity of the sample and significantly reducing equipment costs [99]. Compared to thermally driven systems, acoustic droplet generators offer faster response times (within microseconds) and stronger control capabilities, while generating minimal heat throughout the process, ensuring no adverse effects when integrated with other modules [100]. Compared to mechanical drive systems, acoustic strategies feature a compact and reliable structure with particularly significant advantages in miniaturization [101], and they possess dynamic adjustment capabilities, greatly enhancing flexibility.

### 3.2. Acoustic-Powered Droplet Sorting

#### 3.2.1. Sorting Principles and System Architecture

Cell-loaded droplet sorting technology is a cutting-edge, interdisciplinary microfluidic technique that has emerged in recent years, opening a new avenue for anticancer drug screening, antibody engineering, cell therapy development, and other advanced biological research. Droplet sorting technology is a complex system that integrates microfluidic droplet generation, cell loading, and high-throughput sorting [102]. As mentioned before, the occupation ratio of single-cell droplets is inadequate for statistical analysis, which needs high precise purification to remove undesired droplets. After purification and enrichment, which we call sorting, these fragmented and homogeneous microdroplets can be prepared for a series of biochemical analyses [103]. A typical sorting process contains two steps: sorting target identification and signal-triggered sorting. Before sorting, a reporter system will convert the biological or biochemical signals, e.g., fluorescence, into electrical signals. Then the identification system instantly analyzes and evaluates the signal intensity and ratio of each droplet based on predefined logic. Once a droplet is detected as meeting the preset sorting criteria, the sorting system is immediately triggered, precisely diverting the target droplet into the collection channel, while non-target droplets that do not meet the criteria flow directly into the waste reservoir [104]. Sorted droplets can thus be isolated and used for downstream experiments such as amplification, sequencing, and in-depth analysis.

#### 3.2.2. Comparative Analysis of BAW, TSAW, and SSAW Sorting Platforms

Acoustic-powered droplet sorting can be categorized into three types: BAW sorting, TSAW sorting, and SSAW sorting [85].

**Figure 4:**
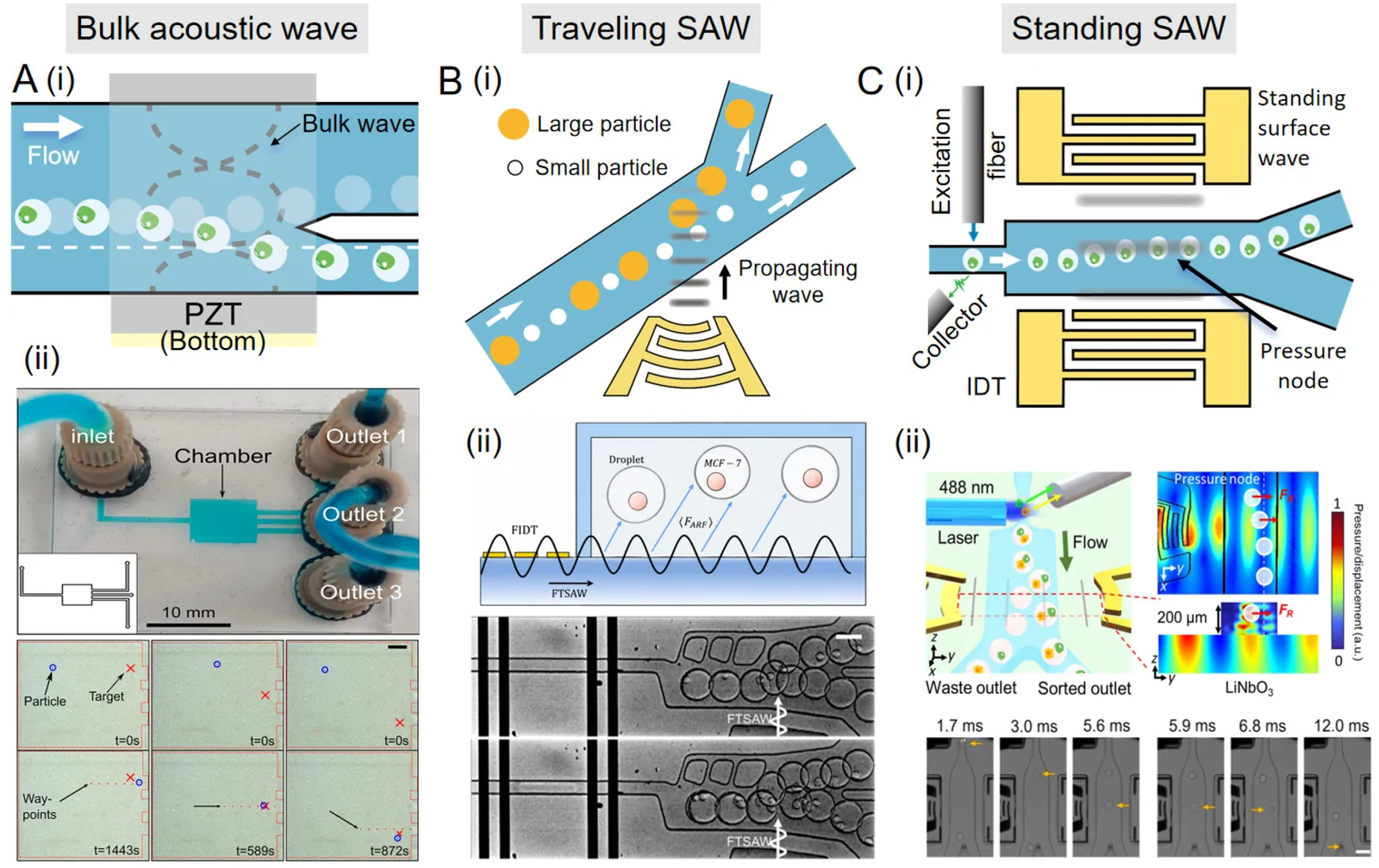
A diagram showing the principle and categories of acoustic-powered droplet sorting methods. (**A**) BAW sorting mechanism (**i**) and its application (**ii**) [107]; (**B**) TSAW sorting mechanism (**i**) [120] and its application (**ii**) [110]; (**C**) SSAW sorting mechanism (**i**) and its application (**ii**) [117].

BAW droplet sorting utilizes the mechanical effects generated when acoustic waves propagate through the fluid [49,59]. When a droplet containing target cells flows through the acoustic field, differences in density and compressibility between the droplet and the surrounding fluid cause uneven forces on the droplet’s surface, pushing it toward a specific stable position within the acoustic field (usually the pressure node) (Figure 4A(i)). In 2014, L. Schmid et al. [105] developed a BAW-based microfluidic fluorescence-activated sorter incorporating an optical detection region, enabling high-speed, high-throughput droplet sorting and supporting the sorting of droplets into two or more outlet channels. In 2015, L. Leibacher et al. [106] further systematically explored the diverse application capabilities of BAW in microfluidic droplet handling, comprehensively demonstrating the various possibilities of BAW technology for droplet manipulation. They integrated a series of operations—including the coalescence, focusing, sorting, and medium exchange of 200-micrometer water-in-oil droplets—onto a single platform. In 2021, K. Yiannacou et al. [107] built on these advancements by combining BAW with machine learning, achieving precise, automated two-dimensional manipulation and active sorting of droplets within a microfluidic chip (Figure 4A(ii)). This approach addressed the challenge posed by traditional methods, where a single acoustic transducer alone could not provide flexible positional control over individual particles.

Compared to BAW sorting, TSAW sorting offers higher accuracy and integration [25,26]. When a radiofrequency signal of a specific frequency is applied, the photolithographically fabricated IDT can excite acoustic waves that propagate unidirectionally along the surface of the piezoelectric substrate [100,108]. The energy of these waves is almost entirely localized within a depth of one wavelength at the substrate surface, forming an excitation zone that propagates along the surface (Figure 4B(i)). When a fluid flows through this region, the acoustic energy leaks into the fluid, converting kinetic energy into fluid momentum [109]. In 2023, J. Zhong et al. [110] combined TSAW with electrical detection, first using electrical impedance to rapidly screen droplets and then employing an acoustic method to efficiently sort the selected single-cell droplets (Figure 4B(ii)). E. S. Richter et al. [111] took a different approach by combining optical absorbance detection with TSAW, achieving high-speed droplet sorting at the kHz level and effectively enhancing detection sensitivity through an integrated lens-based fiber-optic interrogation system. Also in 2023, M. Ali et al. [112] focused on purely acoustic sorting methods, leveraging differences in acoustic impedance between droplets caused by variations in chemical composition to achieve label-free, detection-free sorting. In 2024, they proposed a TSAW-based acoustic streaming control method for sorting target droplets containing cells or particles from empty droplets [113]. Droplets encapsulating samples are subjected to greater acoustic radiation forces, resulting in different lateral migration distances along the TSAW direction compared to empty droplets, ultimately achieving spatial separation. By 2025, building on their previous research, M. Ali et al. [114] further developed the TSAW method by co-encapsulating polymer carrier particles within the droplets to amplify the acoustic differences between cell-containing and cell-free droplets, thereby significantly improving separation efficiency.

SSAW sorting combines the advantages of BAW and TSAW. It is a technology that enables precise static manipulation on a planar surface [19,115]. Its excitation mechanism is similar to that of TSAW: by simultaneously exciting two sets of oppositely placed IDTs, or by using a single transducer in conjunction with carefully designed reflective boundaries, two columns of TSAW propagating in opposite directions can be made to interfere, ultimately forming a completely stationary SSAW with a periodically distributed amplitude on the substrate surface (Figure 4C(i)) [19,116]. In this field, T. J. Huang’s team has long been a pioneer and leader. In 2013, the team first demonstrated a multi-channel on-chip droplet sorter based on tunable SSAW [19]. By adjusting the frequency of the input signal to change the wavelength of the acoustic waves excited by the IDTs, they continuously shifted the spatial position of the acoustic pressure nodes, enabling picoliter-scale droplets to follow the moving nodes and be guided to up to five different outlet channels. In 2021, the team proposed and validated a novel acoustic streaming-controlled droplet sorting chip based on single-phase focused transducers (SPFTs) [16]. By utilizing the focused acoustic field generated by SPFTs to precisely manipulate droplets, they enabled the chip to operate with an input voltage of less than 20 Vpp—far lower than the over 200 Vpp required by many traditional devices—significantly reducing energy consumption and system complexity. In 2023, the team expanded their research to complex biological applications, developing a modular acoustic streaming control platform called CIAMAP [117]. Using SSAW technology, they achieved efficient and precise cell pair droplet sorting and real-time monitoring of the dynamic process of immune response formation (Figure 4C(ii)). The CIAMAP provides a powerful tool for high-throughput single-cell co-culture and immunotherapy research.

Acoustic droplet sorting demonstrates exceptional performance in terms of biocompatibility and manipulation precision. It relies entirely on the cells’ own physical characteristics for manipulation, without the addition of any reagents that might interfere with cell viability or function, thereby preserving the cells’ original integrity to the greatest extent possible—a critical factor for subsequent functional T-cell screening and organoid culture [35,114]. In terms of manipulation scale, with the aid of SAW and BAW, the acoustic field can generate fine-tuned force gradients ranging from single cells at the micrometer scale to exosomes at the nanometer scale, making the efficient separation of minute, precious samples no longer a challenge [118]. Furthermore, this technology offers powerful compatibility. It is not only compatible with open microfluidic chips but can also be seamlessly integrated with modules such as optical imaging and electrical impedance detection, making it highly suitable as the core driving unit for constructing multifunctional ‘lab-on-a-chip’ systems [119].

### 3.3. Acoustic Droplet Manipulation

**Figure 5:**
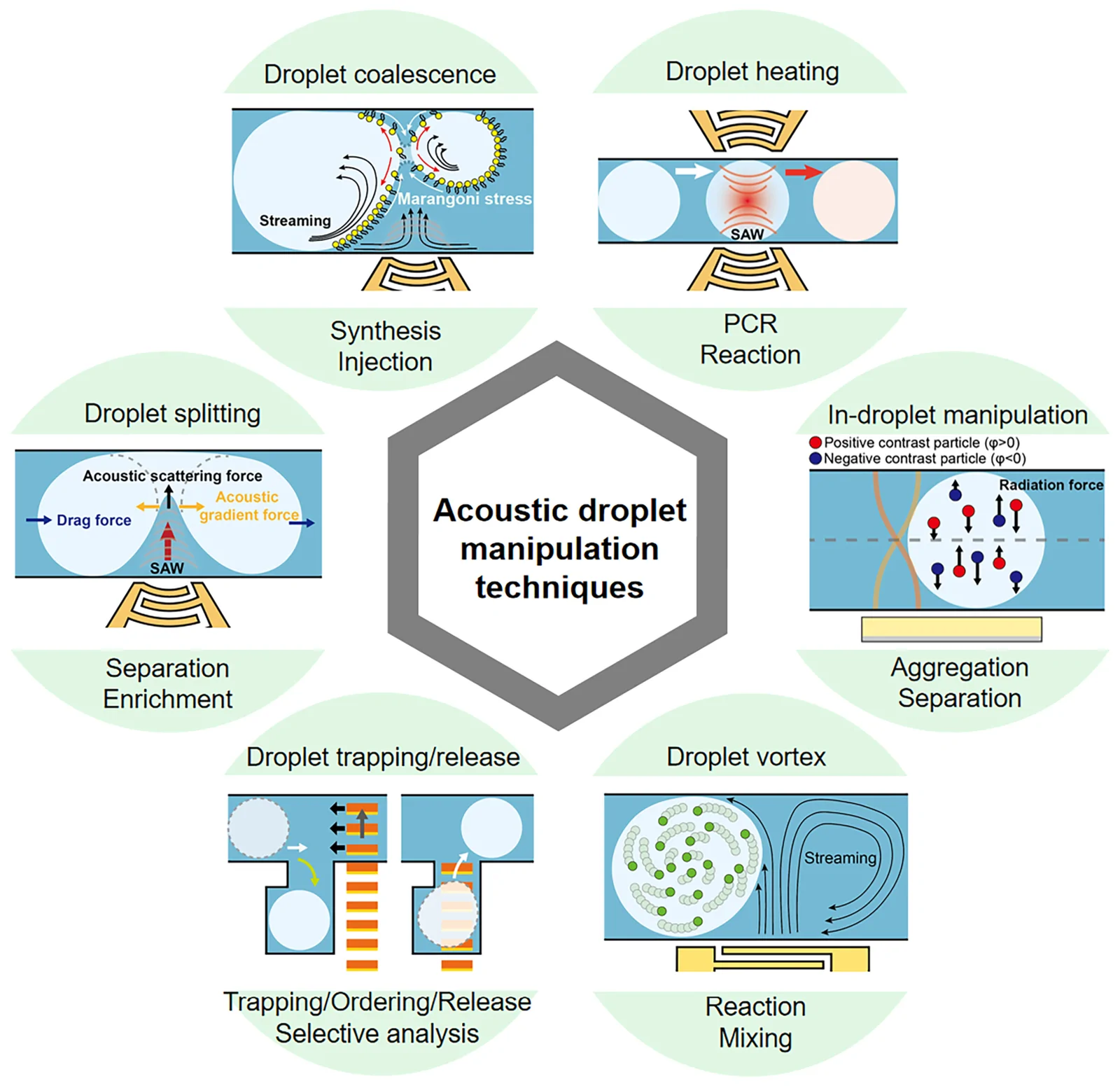
A diagram showing the categories and main applications of acoustic droplet manipulation methods.

Acoustic droplet manipulation is another major application of acoustic waves apart from droplet sorting [85]. Acoustic droplet manipulation technologies utilize specific designs and structures to generate and focus high-frequency acoustic waves, causing them to propagate along a predetermined path and thereby establish a controllable force field with high accuracy [121,122]. This force field can overcome the effects of liquid surface tension, viscosity, and gravity to push, pull, or trap droplets, thereby enabling complex operations such as splitting, merging, and vortexing [122,123]. Depending on the target of action, acoustic manipulation can be divided into manipulation of droplets and manipulation inside droplets (Figure 5). Compared to traditional methods that rely on physical tubes, pipettes, or micropumps, acoustic manipulation enables true non-contact operation, completely eliminating sample cross-contamination and loss caused by adsorption and residues on pipette tips or tube walls. This makes it particularly suitable for handling precious samples or toxic substances [123]. It also offers exceptional precision and flexibility, capable of easily handling volumes ranging from picoliters to microliters. Moreover, it can dynamically alter droplet trajectories in real time to perform complex manipulations that are difficult to achieve with conventional methods [85]. Most importantly, it possesses significant integration potential, making it suitable for the development of highly integrated, automated chips [108].

#### 3.3.1. Manipulation of Droplets: Splitting, Merging, Trapping and Release

The ability to treat a droplet as a unified entity is essential for advanced droplet operations. The basic manipulations include moving, splitting, merging, trapping, and releasing. These manipulation methods ensure that the droplet, as a unit, can be flexibly utilized for diverse applications on demand.

**Figure 6:**
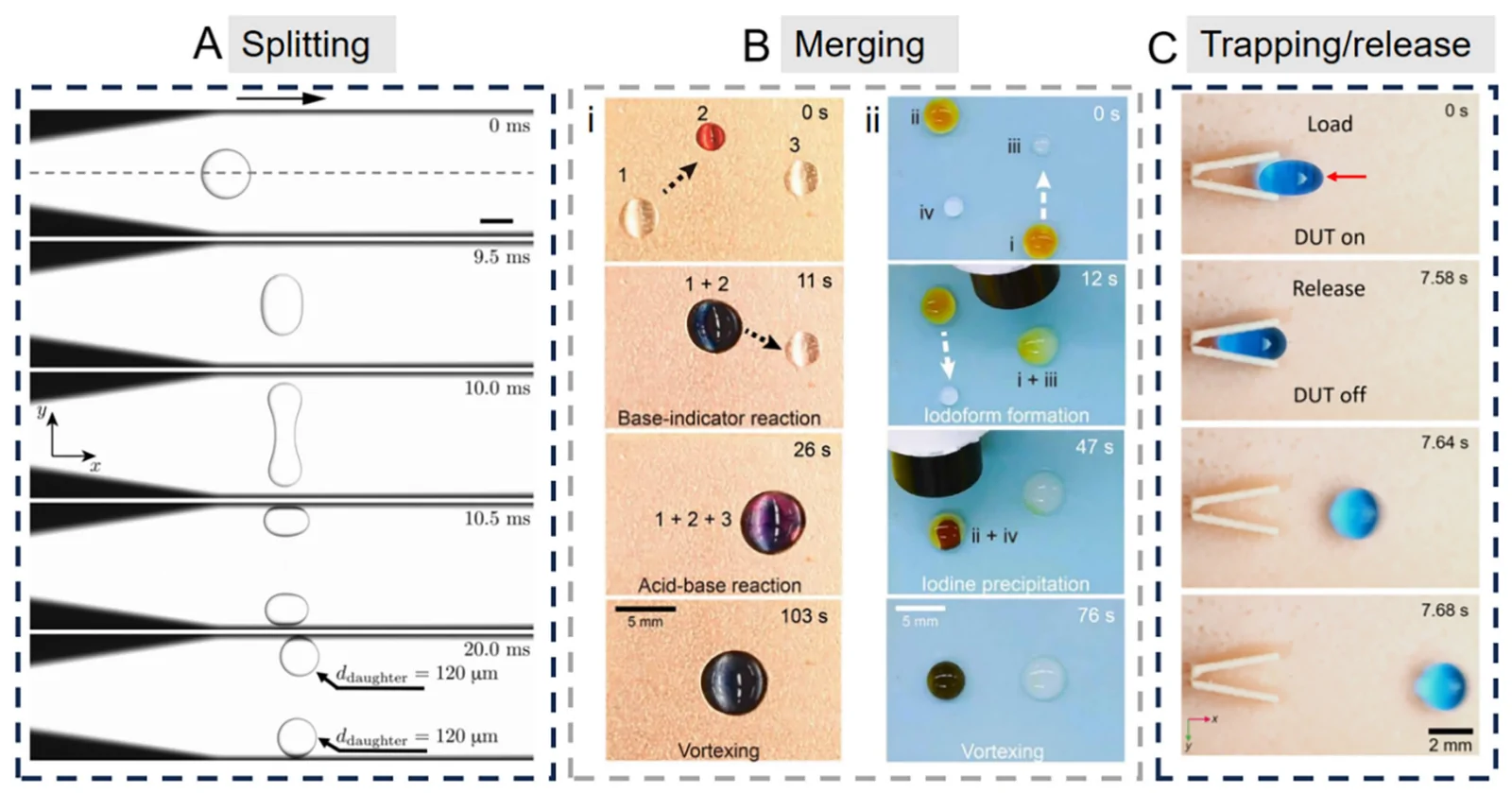
A diagram showing acoustic droplets splitting, merging, trapping and release. (**A**) Acoustic droplet splitting, droplets splitting proportionally caused by a standing-wave field [125]. (**B**) Acoustic droplets merging on the copper substrate (**i**) and on the nitrile rubber substrate (**ii**); NaOH droplets i and ii react respectively with acetone droplet iii and HCl droplet iv to produce an iodoform suspension and iodine precipitate [127]. (**C**) Acoustic droplet trapping/release: the droplet gets squeezed into the crack and then released [131].

In the field of acoustic droplet splitting, in 2024, M. Sui et al. [124] published a study on acoustic micro-tweezers, integrating droplet actuation and impedance sensing onto a single platform for the first time, thereby achieving automated closed-loop control of operations such as droplet transport, merging, splitting, and mixing. The system integrates orthogonally arranged oblique IDTs and a custom-designed control and detection circuit system, enabling not only selective actuation of specific droplets but also simultaneous sensing of changes in droplet position and volume. Through feedback control, the system achieved precise splitting of 2-microliter droplets, with the volume deviation of the split droplets controlled within 5%. In 2025, D. Xu et al. [125] proposed a single-frequency, one-dimensional standing-wave acoustic-fluidic technique for high-throughput continuous control. By utilizing the symmetric effect of bidirectional acoustic radiation pressure exerted on droplets at acoustic pressure nodes, they achieved continuously adjustable droplet splitting within the channel, with the entire splitting process taking only 1 ms (Figure 6A). Experimental data show that this technology can maintain proportional splitting at flow rates as high as 161 microliters per minute, achieve non-proportional splitting at lower flow rates, and is compatible with a variety of droplet systems. In 2026, W. Wei et al. [126] published research on “acoustic nanoscissors,” which extends the operational scale of acoustic splitting down to nanometer and femtoliter levels. By exciting the transverse vibration mode of a high-frequency BAW, they generated a concentrated shear stress field on a thin liquid film and used a non-contact method to precisely shear a continuous fluid stream into discrete femtoliter-scale droplets. Compared to other existing acoustic methods, this platform represents a reduction of more than three orders of magnitude. Furthermore, by controlling the phase and amplitude of the acoustic field, this platform can not only generate ordered droplet arrays with specific patterns but also switch between droplet splitting and coalescence within the same system.

In addition to splitting, multiple droplets can be merged as well. In 2025, H. Zhu et al. [127] spatially coupled an acoustic swimming force field with a super-slip surface to construct a tunable acoustic swimming force field. This system can manipulate a wide variety of droplets—ranging from extremely large volumes to those with a broad range of surface tensions—under non-contact, non-destructive conditions. By modulating the acoustic swimming force field, it enables two residue-free merging modes: thrust-driven extrusion and pull-driven convergence. The addition of a sound-controlled fluidic processor (SFP) makes on-site colorimetric reactions, precipitation reactions, and biosensing feasible (Figure 6B(i,ii)). Moreover, SFP has successfully enabled the culture and drug testing of primary mouse liver organoids, indicating its powerful versatility. In the same year, J. Li et al. [128] utilized a dual-sided phased array to achieve high-throughput merging of multiple pairs of droplets. They revealed the principle that wide traps are more conducive to droplet merging than narrow traps, and constructed uniform acoustic traps through uniform energy distribution, significantly increasing the number of droplets that can be suspended simultaneously. To address the challenges of designing acoustic fields for multiple traps, the same team also proposed the global algorithm direct search phased array technology (DS-PAT). Based on this method, for the first time, simultaneous and stable merging of up to six pairs of droplets was achieved. Building upon this, the research further designed a sequential merging scheme for four columns of droplets and successfully applied it to the Belousov–Zhabotinsky (BZ) oscillatory reaction, demonstrating its practicality in chemical reactions involving the stepwise addition of multiple reagents. In 2026, Z. Yuan et al. [129] published research on the droplet ultrasonic tweezer (DUT), further enabling the programmable coordinated manipulation of multiple droplets and expanding their spatial degrees of freedom. They employed 28 ultrasonic transducers to form a double-layer annular phased array, which was used to generate a twin-trap acoustic field. This acoustic field can stably confine droplets at five specific positions around the focal point, enabling the programmed transport of up to three droplets simultaneously. It also allows for asynchronous transposition and merging of droplets through a dynamic vortex induced by the acoustic field. In the vertical direction, the acoustic field possesses extensibility, capable of simultaneously driving droplets on two surfaces separated by 6 mm. The DUT accelerates the droplet merging rate while preventing the merged droplet from detaching from the substrate. By increasing the size of the detached droplet and expanding the surface-cleaning range, it enables more efficient surface renewal.

In the field of acoustic droplet trapping and release, in 2021, X. Qin et al. [130] utilized two focused acoustic fields in a microfluidic channel to construct a switchable virtual barrier, thereby pioneering the concept of an “acoustic valve.” This valve enables non-contact capture and release of droplets via acoustic radiation forces: when open, it can precisely block specific droplets and guide them to a target branch channel; when closed, it immediately releases the captured droplets, allowing them to continue flowing downstream. The entire process requires no sample labeling or modification and avoids damage caused by high-voltage electric fields. In 2023, Z. Yuan et al. [131] shifted their focus to open superhydrophobic surfaces and proposed a DUT system based on an ultrasonic phased array. They constructed a dual-trap acoustic field to capture droplets and drove the droplets along arbitrarily programmed paths by altering the position of the acoustic field’s focal point, thereby freeing acoustic manipulation from prerequisites such as surface pretreatment or droplet modification. As the droplet moves, it is subjected to an unbalanced acoustic radiation force, which moves it to a pressure node where it is stably trapped. By altering the position or intensity of the acoustic field’s focal point, the balance between the acoustic radiation force and the constraint forces acting on the droplet can be disrupted, enabling on-demand release of the droplet (Figure 6C). In 2025, S. Liu et al. [132] proposed an acoustic demoting mechanism applicable to the biomedical field. This technology focuses high-frequency ultrasound within the droplet, utilizing reflections at the gas–liquid interface to form a standing-wave field, thereby creating an extreme pressure gradient within the droplet itself. This inward-to-outward force is numerically three orders of magnitude greater than that of conventional acoustic streaming forces. During capture, because the driving force overwhelmingly exceeds the capillary adhesion forces of super-hydrophilic surfaces by several orders of magnitude, droplets can be stably captured without slipping or escaping, even on surfaces with extremely low contact angles and strong pinning effects. Upon release, since the capture force originates from acoustic field confinement within the droplet rather than a change in interfacial properties, the internal stresses in the droplet disappear instantaneously once the acoustic field is turned off, leaving no residual force field or interference from surface modifications between the droplet and the substrate.

#### 3.3.2. Manipulation Inside Droplets: Heating, Vortex, and Particle Manipulation

While droplet-level manipulation provides fundamental controls over the entire droplet, tighter regulation of internal components greatly enhances the functionality and versatility of acoustic droplet manipulation. Current in-droplet acoustic manipulation techniques can be categorized into three branches: heating, vortex, and in-droplet particle manipulation. The well-arranged arrangement of different droplet manipulation methods enables versatile applications such as precise microreactions, on-chip synthesis, cellular reactions, and so on.

**Figure 7:**
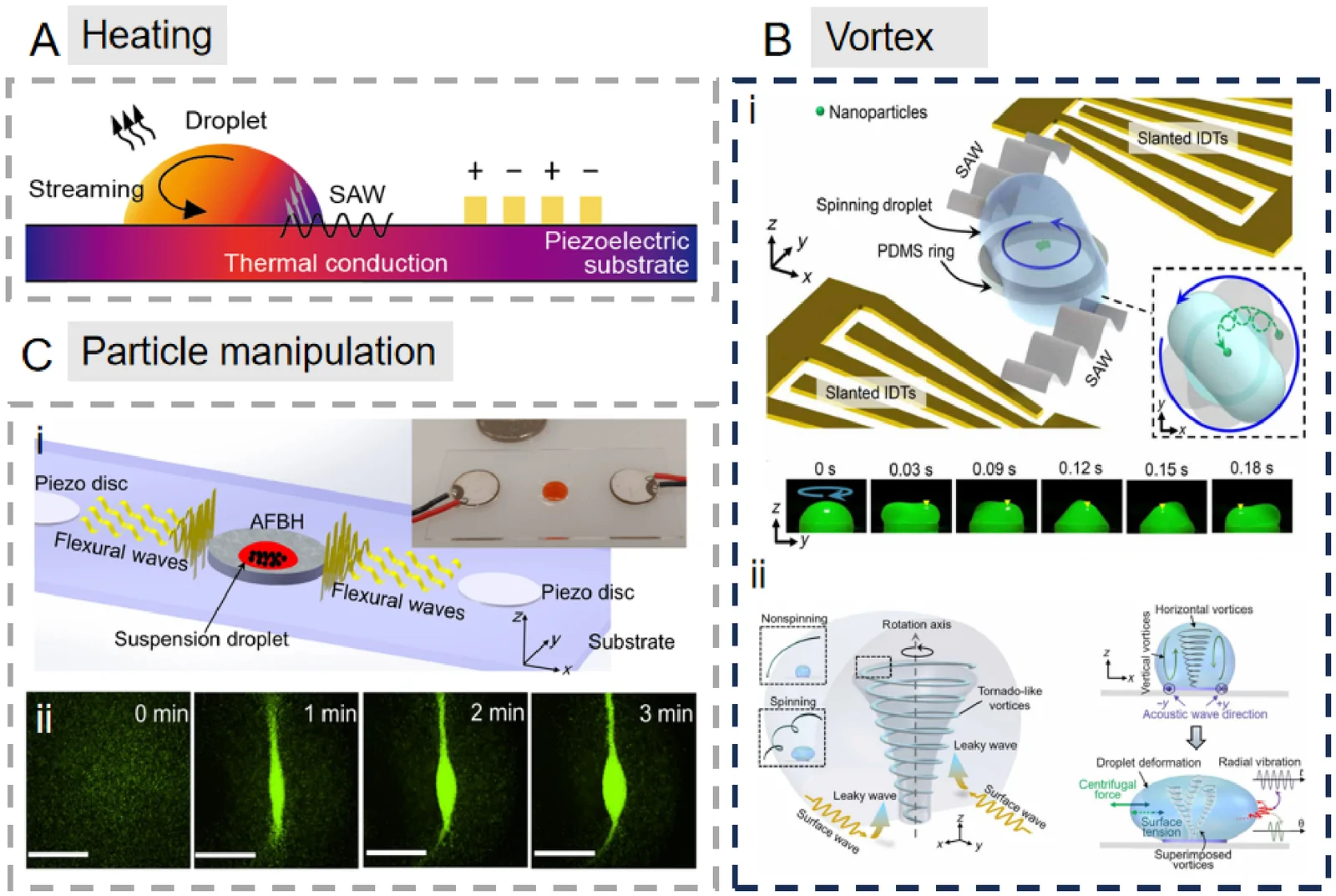
Mainstream applications of acoustic manipulation inside droplets. (**A**) Acoustic droplet heating. The basic principle of heating a droplet using a SAW-based microfluidic device [133]. (**B**) Acoustic droplet vortex. Top view (**i**) [118] and side view (**ii**) [137] schematics show the functionality of an acoustofluidic in-droplet centrifugation system. (**C**) Acoustic in-droplet particle manipulation. A schematic of a dual-transducer device based on AFBH (**i**) and the gradual accumulation of 10-micron fluorescent polystyrene particles at the acoustic anti-node in AFBH (**ii**) [139].

In the field of acoustic droplet heating, in 2021, L. Li et al. [133] established a theoretical model of the acoustic-thermal processes in a droplet located along the path of a traveling wave, elucidating the mechanism of acoustic-thermal heating of the droplet driven by SAW and exploring methods for controlling its temperature field (Figure 7A). They pointed out that the acoustic-thermal effect generated by electro-mechanical-acoustic coupling is the dominant heat source for droplet heating, while acoustic vortices induced within the droplet by the nonlinear effects of the incident wave—reaching velocities as high as 20 mm/s—trigger forced convection, significantly enhancing internal heat transfer. The equilibrium temperature field of the droplet is ultimately determined by the combined effects of dissipative acoustic attenuation and acoustic streaming. In 2023, K.N. Nampoothiri et al. [134] began exploring practical applications of the acoustic-thermal effect, utilizing the synergistic effects of acoustic heating and acoustic streaming for microdroplet de-icing. Using an IDT as the excitation device, this work systematically observed the dynamic process of SAW from excitation to complete de-icing. The nanoscale mechanical vibrations excited by the SAW at the solid–liquid interface, combined with localized heating, weakened the adhesion between the ice and the substrate, causing the ice layer to detach first. Subsequently, the acoustic streaming effect in the residual liquid water within the droplet was triggered, immediately forming intense vortices. This led to a sharp increase in heat transfer efficiency, as well as rapid rises in temperature and the proportion of liquid water, propelling the entire de-icing process into a high-speed phase. In 2025, L. Huang et al. [135] driven by the need for integrated development, designed a digital polymerase chain reaction (PCR) chip based on acoustic-thermal droplet heating, employing an array of SAW heating devices for microdroplet reaction systems. The physical chip employs a SAW microfluidic heating platform based on polydimethylsiloxane (PDMS) chambers, capable of simultaneously applying millisecond-scale rapid heating to multiple detection droplets, with no additional heat-conducting components between the heating module and the sensor chip. By adjusting the input power, the chip can achieve a controlled temperature rise within the droplets, making it suitable for temperature-sensitive biological assays such as enzymatic reactions and nucleic acid amplification.

Similar in mechanism to acoustic heating, acoustic vortex aims to rearrange internal components and physical properties of droplets. In 2021, Y. Gu et al. [118] pioneered the development of acoustic streaming-controlled centrifugation technology, coupling SAW-driven droplet vortices with acoustic streaming effects to construct a non-contact micro- and nano-scale centrifugation platform (Figure 7B(i)). They utilized SAW to induce spiral vortices within the droplet and drove the droplet’s overall vortex via acoustic radiation pressure. The Stokes drift effect within the droplet amplified both the shear rate and the flow velocity, enabling efficient enrichment and size sorting of nanoparticles within one minute, which is far faster than traditional centrifugation methods. In addition, the system shows the capability of the clean and precise separation of exosome subpopulations, with a minimal sample volume of five microliters, surpassing sample volume restrictions of conventional centrifugation methods. In 2024, X. Qin et al. [136] utilized a movable surface acoustic wave tweezers system to achieve both in-plane and out-of-plane vortex modes for droplets within microchannels. This system can drive droplets to perform continuous, controllable in-plane vortices around any point within a plane, while also enabling three-dimensional vortices of droplets along an out-of-plane axis; the vortex speed and angle can be flexibly controlled by adjusting the movement parameters of the substrate. In 2025, C. Chen et al. [137] reported the precise control mechanism for three-dimensional manipulation of particles within acoustic-controlled droplets (Figure 7B(ii)). They systematically analyzed the relationship among acoustic field parameters, droplet properties, and particle trajectories, discovering that the flow field induced by SAW in the equatorial region of the droplet forms rotating Stokes waves. When fluid motion couples with these waves, a series of controllable superimposed helical trajectories are generated. In the experiments, particle velocities reached 2 cm/s, marking the first successful achievement of fully three-dimensional trajectory control of particles within an acoustically controlled droplet.

Advancing from the acoustic vortex, acoustic in-droplet particle manipulation provides a direct internal component rearrangement solution. In 2017, K. Park et al. [138] proposed a method for size-graded separation of particles within droplets based on TSAW, generated by novel slanted finger interdigital transducers (SFITs). They applied traveling-wave acoustic fields generated by two transducers operating at different frequencies sequentially to a moving water-in-oil droplet, first simultaneously propelling 5-micrometer and 10-micrometer particles to complete pre-enrichment, and then using low-frequency acoustic waves to selectively deflect the larger particles. This platform achieved a differentiated lateral distribution of the two particle sizes within the same droplet, with separation ultimately completed as the droplet flowed through a Y-shaped fork, yielding separation efficiencies of 86.3% and 86.9%, respectively. In 2022, P. Liu et al. [139] proposed the concept of an “acoustic streaming-controlled black hole” and designed a microstructure platform with a gradient thickness. They enabled acoustic waves emitted by piezoelectric transducers to be effectively captured and compressed by the acoustofluidic black hole (AFBH) structure, thereby forming a highly localized, intense acoustic field within the droplet to achieve various manipulations of particles inside the droplet (Figure 7C). Experimental results demonstrated that the AFBH platform can achieve particle translation, concentration, and patterned arrangement within a single droplet, exhibiting outstanding multifunctional integration capabilities. In 2023, L. Huang et al. [140] published a study on the nanoparticle enrichment process in acoustic microfluidic systems, focusing on nanoscale targets. They proposed a PDMS film with a microporous structure to immobilize and drive the droplet into vortex motion under acoustic field excitation. Based on the moving mesh method, they established a coupled finite element model of the droplet’s dynamic deformation and the evolution of its internal flow field, systematically investigating the motion trajectories and enrichment patterns of particles under the synergistic effects of vortex and deformation. The results indicated that the dynamic deformation of the droplet in the acoustic field is the key mechanism for inducing effective nanoparticle enrichment. In 2025, M. Du et al. [119] focused on the critical technical challenge of rapidly separating particles within immobilized droplets and proposed a simplified SAW platform. By leveraging the synergistic effects of acoustic streaming forces and acoustic radiation forces, they achieved precise, non-contact manipulation of particles within immobilized droplets. By varying the drive frequency and positional offset factor, the researchers experimentally observed, for the first time, a steady-state condition in which a central enrichment cluster coexists with concentric outer rings, revealing the dynamic equilibrium mechanism whereby particles are simultaneously subjected to both centripetal radiation forces and centrifugal acoustic streaming forces within the acoustic field.

## 4. Perspective

### 4.1. Challenges and Opportunities in Acoustic-Controlled Droplet Generation

While individual acoustic control units can now achieve high-speed droplet generation, it remains extremely challenging to achieve large-scale parallel droplet generation. Arranging a large number of acoustic transducers within a limited chip area not only faces physical constraints on spatial layout but also requires addressing crosstalk and interference between adjacent acoustic field units. Furthermore, when the system is scaled from a single channel to multi-channel parallel operation, ensuring consistency in the acoustic field across channels and uniformity in droplet generation remain challenging. Machine learning and artificial intelligence algorithms could be introduced to optimize acoustic field parameters in real time, potentially enabling the prediction and control of droplet generation parameters within milliseconds, thereby ensuring stable droplet output even under fluctuating fluid conditions. Alternatively, acoustic metamaterials and microstructure arrays could be utilized to locally modulate the acoustic field, potentially enabling the parallel operation of a large number of independent acoustic control units on a single chip.

Another challenge accompanying large-scale parallel design is the integration of the acoustic module with the microfluidic module. A detachable dual-chip architecture has already been proposed. Future research could focus on separating SAW devices from disposable microfluidic chips to ensure the stability of core component reuse and further reduce the cost of disposable consumables. Additionally, introducing novel piezoelectric films and flexible substrates could extend acoustic droplet generation to wearable flexible platforms, eliminating reliance on inorganic piezoelectric materials and broadening the technology’s scope of application.

### 4.2. Challenges and Opportunities in Acoustic-Powered Droplet Sorting

Insufficient acoustic contrast among similar targets is the major drawback that hinders the development of acoustic droplet sorting. Label-free acoustic sorting relies on the acoustic contrast between target particles and the surrounding medium. When cells of different types are encapsulated within droplets but have similar acoustic properties, purely acoustic methods struggle to distinguish them effectively. Although current approaches involve co-encapsulating specific polymeric particles to elicit an acoustic response, the pretreatment steps can still affect precious samples such as primary cells, and the experimental protocol requires further optimization. Integrated multimodal real-time detection systems can overcome the bottlenecks of conventional cell sorting; however, integrating high-speed bright-field imaging, fluorescence detection, and even impedance sensing with acoustic sorting onto a single platform is overly complex. Future directions may rely on machine learning algorithms, which can be introduced to automate the real-time processing and decision-making of this multimodal information, enabling sorting parameters to be dynamically adjusted within milliseconds based on sample characteristics.

As a relatively new technology, acoustic droplet sorting lacks industry-recognized, unified standards for hardware specifications, chip manufacturing, and operational procedures across different laboratories. This lack of standardization has significantly slowed the cross-platform adoption and commercialization of the technology, and it also makes it difficult to directly compare performance metrics between different research groups. The industry urgently needs to establish unified standards across three key areas: device architecture, interface specifications, and performance evaluation systems. The development of more detachable, modular acoustic sorting systems and standardized acoustic manipulation modules will significantly lower the barriers to entry and reduce costs, while also enhancing interoperability and reproducibility across different platforms.

### 4.3. Challenges and Opportunities in Acoustic Droplet Manipulation

Manipulation of submicron and nanoscale particles: As the scale of acoustic manipulation targets shrinks from the micrometer to the nanometer range, the effects of acoustic radiation forces decay rapidly, while the random effects of Brownian motion become increasingly pronounced. This makes it more difficult to stably trap and precisely guide particles within an acoustic field. Within droplets, although acoustic vortices can achieve large-scale convection, it is difficult to establish spatially selective steady-state trapping potentials at the nanoscale. Currently, the synergistic use of GHz-level ultra-high-frequency acoustic waves and acoustic microstructures has reestablished the dominance of acoustic forces over Brownian motion, opening up the exploration of precise manipulation of sub-100 nm biological particles. Meanwhile, acoustic metamaterials and gradient-thickness films can localize the acoustic field to deep sub-wavelength scales, thereby constructing highly efficient potential wells dominated by acoustic radiation forces at the nanoscale.

Dynamic control of three-dimensional degrees of freedom: Although acoustic levitation has enabled three-dimensional manipulation of droplets in air, the degrees of freedom for three-dimensional droplet motion remain severely limited in substrate-constrained microfluidic chips. Equatorial vortexal waves can drive particles within a droplet to form three-dimensional spiral trajectories, but control over the droplet’s overall longitudinal motion remains inadequate. The ability to independently manipulate the droplet in all three dimensions needs to be enhanced. In the future, the introduction of multi-faceted integrated transducer arrays, or the use of three-dimensional acoustic holography combined with two-dimensional phased array encoding, may enable the generation of asymmetric, dynamically reconfigurable three-dimensional sound pressure distributions within the chip cavity, providing the droplet with a normal acoustic radiation force. Alternatively, integrating on-chip micro-impedance sensing and high-speed confocal micro-imaging technologies with acoustic field actuation could provide real-time feedback on the droplet’s longitudinal position. Machine learning could then be used to optimize the acoustic field in real time, dynamically adjusting the amplitude and phase of multiple transducers, ultimately improving the precision of normal-direction control without requiring hardware modifications.

All in all, acoustics plays a fundamental role in droplet microfluidics, participating in every stage from initial generation and sorting to advanced downstream complicated manipulation and biochemical analyses. This review systematically summarizes the integration of acoustics with droplet technologies. The combination of two techniques opens up substantial opportunities for scientific and engineering studies. With further improvement, applications of acoustic droplet microfluidics can extend beyond high-throughput sorting, on-site sensing, and on-chip drug synthesis and screening, targeting emerging advanced fields such as rare-cell isolation, personalized medicine, environmental monitoring, and more.

## Data Availability

No new data were created or analyzed in this study. Data sharing is not applicable to this article.

## Abbreviations

The following abbreviations are used in this manuscript:

microTAS	Micrometer-scale Total Analysis Systems
SAW	Surface Acoustic Wave
BAW	Bulk Acoustic Wave
IDT	Interdigitated Transducer
TSAW	Traveling Surface Acoustic Wave
SSAW	Standing Surface Acoustic Wave
FSAW	Focused Surface Acoustic Wave
SPFT	Single Phase Focused Transducer
CIAMAP	Cellular Immunity Analysis by a Modular Acoustofluidic Platform
SFP	Sound-controlled Fluidic Processor
DS-PAT	Direct Search Phased Array Technology
BZ	Belousov–Zhabotinsky
DUT	Droplet Ultrasonic Tweezer
PCR	Polymerase Chain Reaction
PDMS	Polydimethylsiloxane
SFIT	Slanted Finger Interdigital Transducer
AFBH	Acoustofluidic Black Hole
